# Eriodictyol Attenuates MCAO-Induced Brain Injury and Neurological Deficits *via* Reversing the Autophagy Dysfunction

**DOI:** 10.3389/fnsys.2021.655125

**Published:** 2021-05-26

**Authors:** Chuanxiang Wang, Zhequan Ma, Zuqiang Wang, Shuping Ming, Yanbing Ding, Sufang Zhou, Hongyu Qian

**Affiliations:** ^1^Department of Neurology, Hubei Provincial Hospital of Traditional Chinese Medicine, Wuhan, China; ^2^Yangxin County Chinese Medicine Hospital, Yangxin, China; ^3^The First Clinical College of Hubei University of Traditional Chinese Medicine, Wuhan, China; ^4^Department of Emergency, Hubei Provincial Hospital of Traditional Chinese Medicine, Wuhan, China

**Keywords:** eriodictyol, autophagy, middle cerebral artery occlusion (MCAO), inflammation, apoptosis

## Abstract

The present study was designed to investigate the protective effect of eriodictyol on MCAO-induced brain injury and its regulation of neural function and to explore the mechanism of its regulation of autophagy in rats. Brain injury was induced by middle cerebral artery occlusion (MCAO) in adult rats and pretreated with eriodictyol (low dose: 20 mg/kg; medium dose: 40 mg/kg; high dose: 80 mg/kg) or saline. Rats in the treatment group had a smaller volume of infarction and improved neurological outcome and reduced the latency to the platform, increased the time spent in the correct quadrant compared to MCAO rats pretreated with saline. ELISA kits results confirmed that eriodictyol reduced the inflammatory response induced by MCAO. The results of apoptosis and proliferation by Nissl staining and immunofluorescence detection indicated that eriodictyol could inhibit apoptosis and promote the proliferation in MCAO rats. The expressions of LC3, ATG5, p62, and Beclin1 were used to evaluate the autophagy, as well as the reversal of the autophagy activator (rapamycin) on the neuroprotective effect of eriodictyol, which suggested that the protective effect of eriodictyol on brain injury may be related to the inhibition of autophagy. In summary, we, therefore, suggested that eriodictyol could reduce the inflammation response of brain injury and inhibit neuroapoptosis, directly affecting autophagy to alleviate brain injury. It will provide theoretical support for eriodictyol in the treatment of ischemic stroke.

## Introduction

Ischemic brain injury is associated with extremely high rates of disability and mortality around the world. Stroke is a fatal medical condition in which broken or blocked blood vessels that suddenly prevent blood to flow to the brain, starving the tissue of oxygen and glucose. Ischemic stroke is considered to be one of the major fatal diseases with high morbidity, mortality, and disability rate in the world (Mavroudakis et al., 2020). Stroke is the leading cause of death among residents worldwide and is expected to increase by 24.9% by 2030 (from 2010; Chavda et al., 2021). At present, there are few therapeutic strategies for ischemic stroke, and the commonly used and effective treatment in clinical practice is t-PA thrombolysis to restore blood supply and alleviate neuronal damage (Ma et al., 2021). However, prevention of stroke and post-ischemia-reperfusion injury repair remains an urgent and unmet need.

Autophagy is a lysosomal-mediated critical process for degrading misfolded proteins and maintaining homeostasis during cellular physiological processes. Under physiological conditions, autophagy plays a key protective role in adapting to nutrient deprivation and clearing damaged organelles (Zhang et al., 2020a). However, some reports have shown that improper autophagy activation may aggravate excessive damage and consumption of the body in cardiovascular diseases (Foglio et al., 2017), cerebrovascular diseases (Li et al., 2020), cancer (Huang et al., 2019), and other diseases. In particular, the interaction between autophagy and apoptosis in ischemic stroke remains unclear. Ao et al reported that JLX001 could induce autophagy to alleviate cerebral ischemia injury by activating the AMPK-ULK1 signaling pathway (Ao et al., 2019). The neuroprotective mechanisms of Ginaton in ischemic brain injury mainly include activation of AMPK pathway induced autophagy, maintenance of mitochondrial homeostasis, and inhibition of apoptosis (Li et al., 2019). On the other hand, He et al. (2019) found that Puerarin inhibited neuronal autophagy and plays a neuroprotective role in cerebral ischemia (Hongyun et al., 2017). Therefore, autophagy is a double-edged sword in the pathological environment of stroke. We investigated the effect of eriodictyol on autophagy induced by stroke to reveal the possible neuroprotective mechanism of eriodictyol.

Rapamycin, an inhibitor of mammalian target of rapamycin (mTOR), could restore cerebral blood flow via regulating NO release in Alzheimer’s disease mice (Lin et al., 2013). The mTOR pathway plays a vital role in numerous physiological functions especially autophagy activation (Wang et al., 2019). Numerous reports have shown that the mTOR pathway is involved in MCAO-induced injury. Chauhan et al. (2011) reported that rapamycin protected against MCAO-induced focal cerebral ischemia in rats. Whereas, studies have shown that mTOR upregulation can also provide neuronal protection during brain injury (Zare Mehrjerdi et al., 2013). Whether upregulating or downregulating the mTOR pathway has a protective effect on neurons in ischemia-reperfusion injury remains controversial. Similar to the role of autophagy, the role of mTOR pathway upregulation or downregulation in I/R injury is still controversial. Specifically, rapamycin activates autophagy in cerebrovascular injury.

Eriodictyol (Etyol), is a flavonoid (3′,4′,5,7-tetrahydroxyflavanone), which naturally exists in fruits and vegetables, especially in lemon (Minato et al., 2003). It is gaining increasing interest for its beneficial biological effects, such as antioxidant, anti-inflammation, anticancer (Lee et al., 2013), and antimicrobial. Miyake et al. (1998) found that lemon ameliorated oxidative stress in diabetic rats significantly. Moreover, eriodictyol has been reported to protect neuron-like cells against oxidative toxicity through the Nrf2/ARE signaling pathway (Lou et al., 2012). Combined with the previous research, we speculate that eriodictyol may attenuate MCAO-induced brain injury and neurological deficits. Hence, the purpose of the present study was to investigate the effects of eriodictyol on the MCAO-induced brain injury and neurological deficits and the probable mechanisms involved with autophagy.

## Materials and Methods

### Animals

A total of 150 male Sprague–Dawley (SD) rats weighing 240 ± 10 g were purchased from Beijing Huafukang Biotechnology Company Limited. The rats were kept in cages (24 ± 2°C) exposed to a 12-h light/dark cycle, and allowed access food and water freely. In order to minimize the number of animals and relieve the pain of rats, all experimental procedures were carried out in accordance with the guidelines of Hubei Provincial Hospital of Traditional Chinese Medicine for the Care and Use of Laboratory Animals and the National Institutes of Health Guide for Care and Use of Laboratory Animals.

### Middle Cerebral Artery Occlusion (MCAO) Model and Drug Treatment

Rats were randomly divided into five groups. The rats in all groups were pretreated for 14 days, and follow-up experiments were conducted at 24 h after reperfusion. Sham groups: rats received surgery without MCAO; MCAO group: rats were subjected to MCAO (saline treatment); Treatment groups: pretreatment by eriodictyol (low dose: 20 mg/kg; medium dose: 40 mg/kg; high dose: 80 mg/kg), the eriodictyol was administered orally by gavage. Rats were subjected to MCAO. Rats were anesthetized with an intraperitoneal injection of pentobarbital sodium (40 mg/kg) and subjected to MCAO as reported with minor modification (Xingyong et al., 2013). Briefly, surgical separation of the right common carotid artery (CCA), internal carotid artery (ICA), and external carotid artery (ECA) resulted in full exposure. The ECA was ligated, and a 4–0 nylon suture with a silicon tip was then inserted through the ECA stump into the ICA, occluding the MCA, approximately 16–20 mm from the insertion point. After 2 h of MCAO, the suture was removed and the blood circulation was recovered. The skin incision was sutured with silk gently. Sham rats underwent the same procedure without occlusion of the MCA.

Next, to investigate the key role of autophagy in the neuroprotection of eriodictyol against MCAO-induced injury, the rats were randomly divided into MCAO, Rapa (MCAO+Rapamycin, autophagy activator), MCAO+Eri80 (MCAO+80 mg/kg eriodictyol), and Rapa+Eri80 (MCAO+Rapa+80 mg/kg eriodictyol; *n* = 10). After pretreatment with eriodictyol or saline for 14 days, rats were given an i.c.v (intra-cerebroventricular injection) injection of rapamycin (30 ng rapamycin dissolved in 10 μl saline) at 20 min before MCAO, respectively.

### Neurological Evaluation

Neurological deficits were tested after 24 h of ischemia, as described previously (Gong et al., 2017). The observers were blind to the design of the experiment. The neurological scores were recorded as follows: (0) no neurological deficit; (1) when the rats were lifted, their paws were not fully extended and their torso was not bent; (2) when the rat is held on a flat surface by its tail but in a normal stationary position, reduce the resistance to pushing or going around to the opposite side; (3) to turn in a spontaneous circle; and (4) the absence of spontaneous movement (severe deficit).

### TTC Staining

Pentobarbital sodium (40 mg/kg) was used to euthanize the animals 24 h after ischemia, eight rats from each group were chosen randomly by staff members. Brains were evenly cut into 2-mm coronal sections. The sections were stained at a 2% solution of TTC (2,3,5-triphenyl tetrazolium chloride) at 37°C for 20 min (Bederson et al., 1986). Briefly, the infarct volume was obtained by multiplying the total infarct areas by the thickness (2-mm) of the sections. The percentage of infarct volume to total brain volume represents the cerebral infarction.

### Morris Water Maze Test

The Morris water maze test was performed in a circular pool (160 cm in diameter, 55 cm in height) filled with water (24 ± 2°C) to a depth of 23 cm, which was located in a quiet room. The pool was conceptually decided into four quadrants (called zones I, II, III, and IV) and a Plexiglas platform (10 cm in diameter) was submerged in zone II such that its surface was 1 cm below the water surface.

On the 1st, 2nd, 3rd, 4th and 5th day following the induction of MCAO, the Morris water maze test was performed as described previously (Wang et al., 2017). Rats were placed into the maze at different starting zone, facing the pool wall. Rats were allowed to find a platform during the 60 s session and were guided to the platform if they failed to find the platform. Rats had to stay on the platform for 30 s before being freed and placed in cages filled with paper towels. The next session started after a 60 s break period. Latency to find the platform and time in the target quadrant were recorded during the acquisition phase. The platform was removed on the probe day (day 5 of the experiment). Each rat was allowed to swim freely in the pool for 90 s and the crossing times through the platform were recorded.

### Immunohistochemistry Staining

Briefly, paraffin sections were subjected to dewaxing and hydration. Before incubation with the primary antibodies, the samples were heat-treated, incubated in 3% H_2_O_2_ in deionized water, and rinsed in PBS. After the antibody (Ki67, 1:200) was added, the samples were incubated at 37°C for 1 h and rinsed with PBS. The goat anti-rabbit IgG was added dropwise, then samples were incubated at 37°C for 1 h, washed with PBS, and stained with DAB. Finally, the sample was rinsed in distilled water, dyed with hematoxylin, dehydrated, cleaned, and sealed. A brown stain on the cell membrane, cytoplasm, and/or nucleus or brown granules indicates cortical neurons. Photomicrographs were taken at 40× magnification under the fluorescent microscope (Leica, Germany). Five non-overlapping views were randomly observed in each section and the positive cell count was performed by experimenters blinded with the aid of ImageJ software.

### TUNEL Staining

After the rats were sacrificed, the brain tissues were isolated. The tissues were fixed with 4% paraformaldehyde for 24 h at room temperature, immersed with 100% ethanol, and xylene for 24 h at room temperature. TUNEL staining was performed using the Colorimetric TUNEL Apoptosis assay Kit (Beyotime Biotechnology). Different random fields were collected by optical microscope and stained images were analyzed by ImageJ software. The number of cells in the cerebral cortex with apoptotic nuclei and total cells was counted. The apoptotic index (AI) = TUNEL-positive cells/total cells × 100%.

### Immunofluorescence Staining

Briefly, the brain tissue was isolated and fixed in 4% paraformaldehyde for 24 h at room temperature, soaked with 100% ethanol and xylene at room temperature for 24 h, and embedded in paraffin wax and sectioned into 7-μm sections. Then the sections were deparaffinized in dimethyl benzene and rehydrated through different concentrations of alcohols. Sections were incubated in sodium citrate buffer (pH 6.0) for 10 min at 80°C. After cooling to room temperature, the sections were washed with PBS three times (7 min each time), followed by blocking for 15 min in Triton X-100 and washed again. Then, PBS containing 10% FBS was incubated for 30 min to block the binding of non-specific antibodies. The sections were subsequently incubated with primary antibodies (LC3, 1:500) overnight at 4°C, followed by incubation with fluorescent-labeled secondary antibody (Alexa Fluor 488 Proteintech Group, China) for 1 h at 37°C. They were again washed with PBS three times. The sections were mounted and visualized under confocal scanning microscopy. At least six images were taken from each rat for analysis. The positive ratio of LC3 = LC3-positive cells/total cells × 100%.

### Nissl Staining

Twenty micrometer slices were soaked overnight in ethanol and chloroform solution (1:1, v/v), and then dehydrated in ethanol groups containing 100% ethanol, 95% ethanol, and distilled water. The sections were stained at 37°C for 20 s in preheated 0.05% (w/v) cresol violet solution. The sections were then quickly rinsed with distilled water and differentiated in hydrochloric acid /95% ethanol (1:50) solution for 10 min. The sections were dehydrated with 100% ethanol, cleared by xylene, and fixed with neutral gum. The stained slices were photographed using a microscope (Leica, Germany), and neuronal cells were counted using the ImageJ software.

### TNF-α, TGF-β1, IL-10, and IL-6 Assay

The prepared serum obtained by prior centrifugation was taken out and the expression level of inflammatory cytokines (TNF-α, IL-6, IL-10 and TGF-β1) in the serum was detected according to the instructions of the kits provided by the company (Quantikine, R&D System, CA, USA). Also, inflammatory factors in brain tissue were examined using ELISA kits. Brains were quickly frozen in liquid nitrogen immediately after removal and kept at −80°C. The brain tissues were homogenized by sonication in an ice-cold 0.01 M PBS and centrifuged for 15 min at 2,500× *g*. The supernatants were collected and stored at −80°C for ELISA detection.

### Western Blotting Assay

The proteins were isolated from cerebral cortex tissue using lysis buffer (Beyotime, Shanghai, China), and the concentrations were measured using a BCA kit (Beyotime, Shanghai, China). The samples were separated by 10% SDS-PAGE and electro-transferred to polyvinylidene difluoride (PVDF) membranes. After blocking with 5% skimmed milk for 4 h at room temperature, the membranes were incubated with primary antibodies (Bax, ab182733; Bcl-2, ab182858; cleaved-caspase-3, ab49822; ATG5, 2630s; LC3, ab51520; Beclin1, 3738s; Ki67, ab16667; p62, ab91526 and β-actin, ab8227, Cambridge, UK) overnight at 4°C. After washing with Tris Buffered Saline with Tween-20 (TBST) three times (8 min each time), the membranes were incubated with secondary antibodies (1:20,000, ZSGB-BIO, Beijing, China) at 37°C for 2 h and then stained with ECL reagent (Thermo Fisher Scientific, Waltham, MA, USA). The gray value of the blot bands was measured by ImageJ software to evaluate the relative protein expression. The proteins are normalized to β-actin.

### Statistical Analysis

The data in our study were presented as mean ± SEM. The statistical analyses were performed using SPSS 22.0 software. One-way ANOVA or* t*-test was used to evaluate Ki-67-positive rate, TUNEL-positive cell rate, neuronal survival rate, and mNSS for different groups. A value of *P* < 0.05 was considered statistically significant.

## Results

### Eriodictyol Reduced MCAO-Induced Brain Injury and Improved Neurological Function

**Figure 1:**
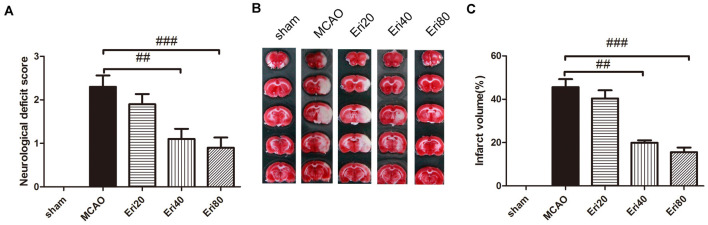
Eriodictyol improved neurological function and reduced middle cerebral artery occlusion (MCAO)-induced brain injury. **(A)** Rats from different groups received either saline or eriodictyol. The neurological score was calculated 24 h after ischemia. ^##^*P* < 0.01, ^###^*P* < 0.001, vs. MCAO group, *n* = 15. **(B)** Representative coronal sections stained with 2% triphenyl tetrazolium chloride (TTC). Magnification: 20×. The white area represents brain infarction. **(C)** Quantification of infarct volume. The data were expressed as mean ± SEM. ^##^*P* < 0.01, ^###^*P* < 0.001, vs. MCAO group, (*n* = 15).

To observe the effect of eriodictyol against MCAO-induced brain injury, rats were treated with different doses of eriodictyol for 14 days before MCAO (Supplementary Figure 1). The neurological score results (Figure 1A) showed that the neurological score in the MCAO group increased significantly compared with the sham group. In eriodictyol treatment group, the neurological score was reduced in a dose-dependent manner markedly with a statistical difference. Brain infarct volume was measured at 24 h after MCAO (Figures 1B,C) by TTC staining. Compared with the sham group, the infarction in the MCAO group was obvious. Compared with the MCAO group, eriodictyol at 40 mg/kg and 80 mg/kg both decreased the infarct volume significantly. But there is no significant difference between the MCAO group and eriodictyol 20 mg/kg. The data showed that eriodictyol could reduce MCAO-induced brain injury and improved neurological function.

### Eriodictyol Ameliorates Cognitive Deficits in MCAO Rats

**Figure 2:**
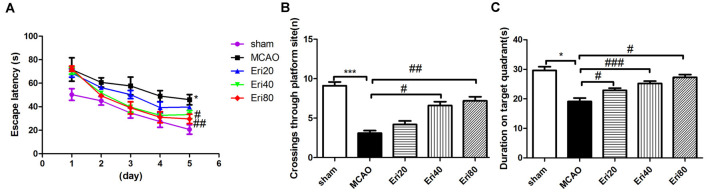
Performance of rats in Morris water maze test. **(A)** The average latencies in sham group, MCAO group, Eri20 group (MCAO+Eri20 mg/kg), Eri40 group (MCAO+Eri40 mg/kg) and Eri80 group (MCAO+Eri80 mg/kg) within 5 days and the mean passes through the target platform **(B)**. **(C)** Mean percentage time in the target guardant during probe trial (The data are presented as mean ± SEM, *n* = 15 each group, *P < 0.05, ***P < 0.001, vs. sham group; ^#^*P* < 0.05, ^##^*P* < 0.01, ^###^*P* < 0.001, vs. MCAO group).

To evaluate the effect of eriodictyol on neural function in MCAO rats, we tested the spatial learning and memory ability of rats by Morris water maze experiment. The results of the Morris water maze experiment (Figure 2) showed that compared with the sham group and the treatment group, the delay time of locating the hidden platform in MCAO group was longer. MCAO group performed poorly on the hippocampal-dependent spatial learning task, while the eriodictyol group showed insufficient resistance to hippocampal-dependent spatial learning tasks to acquire and maintain spatial tasks. The cognitive ability of rats in 40 mg/kg and 80 mg/kg eriodictyol groups was significantly improved.

### Eriodictyol Inhibited the Neuronal Loss and Apoptosis of Brain Tissues in MCAO Rats

**Figure 3:**
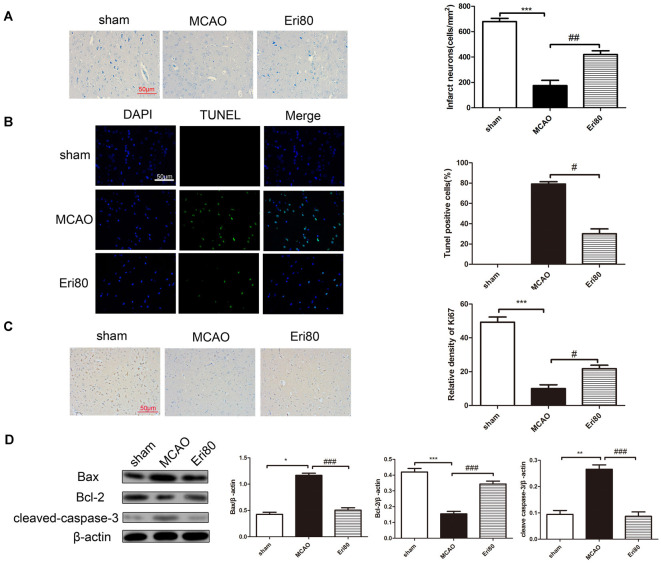
Eriodictyol inhibited the neuronal loss and apoptosis in MCAO rats. **(A)** Effects of eriodictyol on neuronal loss were detected by Nissl staining. **(B)** Apoptosis was evaluated by TUNEL staining. TUNEL-positive signal was shown in green and DAPI was shown in blue. Scale bar: 50 μm. **(C)** Ki67 expression was evaluated by immunohistochemical staining. Scale bar: 50 μm. **(D)** The protein expression were assessed *via* western blotting assay and analyzed by ImageJ software. The bands are represented for the expression of Bax, Bcl-2, and cleaved-caspase-3, which were normalized to β-actin (The data are presented as mean ± SEM, *n* = 3 each group, *P < 0.05, **P < 0.01, ***P < 0.001, vs. sham group; ^#^*P* < 0.05, ^##^*P* < 0.01, ^###^*P* < 0.001, vs. MCAO group).

As shown in Figure 3A, Nissl staining results showed that MCAO could aggravate neural cell loss in the ischemic cortex. Statistical analysis showed that compared with MCAO group, neuron loss was significantly reduced in the treatment group. To examine the effect of eriodictyol on the apoptosis in MCAO rats, the expression of Ki67, cleaved-caspase-3, Bcl-2, and Bax protein were detected by immunochemistry staining and western blotting. TUNEL staining results (Figure 3B) showed that compared with the sham group, the TUNEL positive (green) rate in the MCAO group was significantly increased and the difference was statistically significant. The positive rate was markedly decreased in the treatment group. Immunohistochemical staining results (Figure 3C) showed that the Ki67-positive rate in the MCAO group was significantly reduced compared with the sham group. The positive rate of Ki67 in the treatment group increased significantly. Western blotting results (Figure 3D) showed that the expressions of Bax and cleaved-caspase-3 were significantly increased and the level of Bcl-2 was decreased in the MCAO group compared with the sham group. Whereas pretreatment with 80 mg/kg eriodictyol significantly inhibited this trend.

### Eriodictyol Decreased MCAO-Induced Inflammation

**Figure 4:**
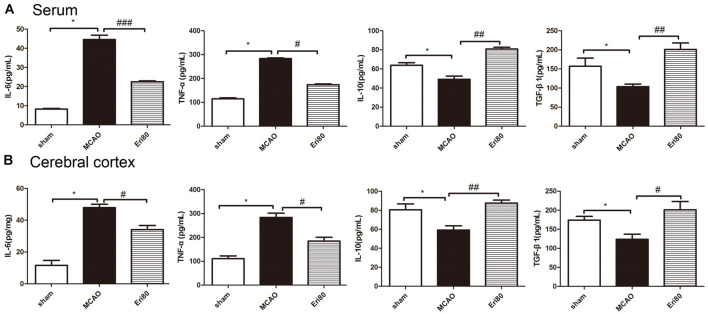
Eriodictyol decreased MCAO-induced inflammation. The levels of IL-6, TNF-α, IL-10, and TGF-β1 in the serum **(A)** and cerebral cortex **(B)** in each group were measured by ELISA kits (The data are presented as mean ± SEM, *n* = 6, *P < 0.05, vs. sham group; ^#^*P* < 0.05, ^##^*P* < 0.01, ^###^*P* < 0.001, vs. MCAO group).

The levels of IL-6, TNF-α, IL-10, and TGF-β1 in serum were detected by ELISA kits. The results (Figure 4A) showed that compared with the sham group, the levels of IL-6 and TNF-α were increased significantly in MCAO group, while the levels of IL-10 and TGF-β1 were decreased significantly. Compared with the MCAO group, the levels of IL-6 and TNF-α decreased markedly in the treatment group, while the levels of IL-10 and TGF-β1 increased. Similarly, the levels of inflammatory cytokines in brain tissue (Figure 4B) showed the same trend as in serum. The data demonstrated that eriodictyol could counteract the changes in pro-inflammatory factors induced by MCAO.

### Eriodictyol Suppressed the MCAO-Induced Enhanced Autophagy

**Figure 5:**
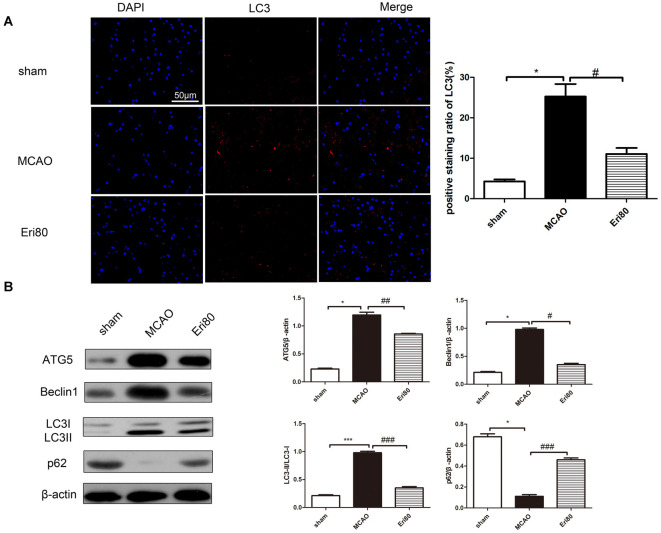
Eriodictyol suppressed the MCAO-induced autophagy. Autophagy activity was assessed by determining the ratio of LC3II/LC3I, the expression of ATG5, p62, and Beclin1 *via* western blotting assay and analyzed by Image J software. **(A)** The protein expression of LC3 was detected by immunofluorescence staining. The LC3-positive signal was shown in red and DAPI was shown in blue. Scale bar: 50 μm (vs. sham group, *P < 0.05, vs. MCAO group, ^#^*P* < 0.05, *n* = 3). **(B)** The expressions of ATG5, Beclin1, p62, and LC3II/LC3I were assessed *via* western blotting assay and analyzed using ImageJ software. Compared with the sham group, the expressions of ATG5, Beclin1, and LC3II/LC3I in the MCAO group were upregulated significantly, while, the expression of p62 was downregulated. After treatment, the protein expressions were reversed (*P < 0.05, ***P < 0.001, vs. sham group; ^#^*P* < 0.05, ^##^*P* < 0.01, ^###^*P* < 0.001, vs. MCAO group, *n* = 3).

Western blotting assay and immunofluorescence staining were performed to detect the expression of autophagy-related proteins and analyze the effect of eriodictyol on autophagy in MCAO rats. As shown in Figure 5A, the positive signal (red) of LC3 was detected by immunofluorescence staining to evaluate the change of autophagy. The results showed that compared with the sham group, the LC3 positive signal was significantly increased in the MCAO group. While the signal was suppressed in the Eri80 group (treatment group). As shown in Figure 5B, the expression of p62 was decreased in the MCAO group, while the expression of ATG5, Beclin1, and LC3 protein conversion was increased. Treatment with 80 mg/kg eriodictyol inhibited the reduction of p62 and increased ATG5, Beclin1, and LC3 protein conversion (*P* < 0.05). The results showed that eriodictyol suppressed the MCAO-induced autophagy.

### The Effects of Eriodictyol on Brain Injury Were Reversed by Rapamycin

**Figure 6:**
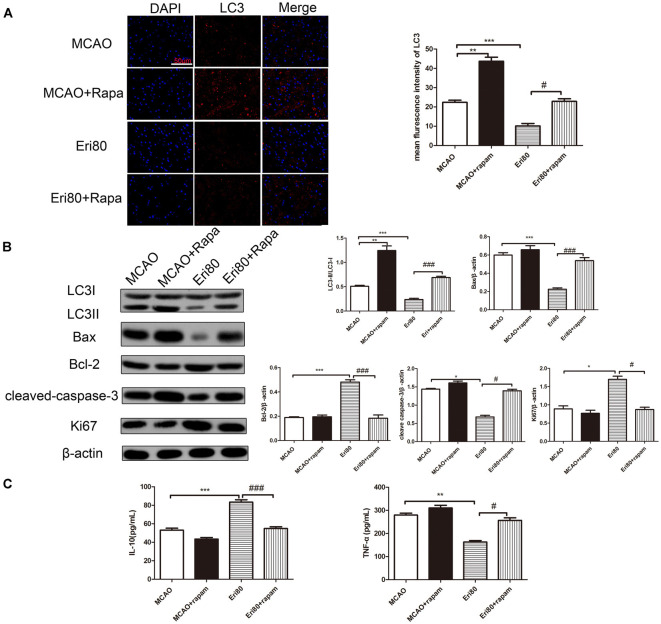
The effects of Eriodictyol on brain injury were reversed by rapamycin. **(A)** The protein expressions of LC3 was detected by immunofluorescence staining. The LC3-positive signal was shown in red and DAPI was shown in blue. Scale bar: 50 μm (vs. MCAO group, ***P < 0.001, vs. Eri80, ^#^*P* < 0.05). **(B)** The protein expression was assessed *via* western blotting assay and analyzed using ImageJ software. The bands are represented for the expressions of LC3, Bax, Bcl-2, cleaved-caspase-3, and Ki67 in different groups, which were normalized to β-actin. **(C)** The concentration of IL-10 and TNF-α in the serum were measured using ELISA kits (The data are presented as mean ± SEM, *n* = 3, *P < 0.05, **P < 0.01, ***P < 0.001, vs. MCAO group; ^#^*P* < 0.05, ^###^*P* < 0.001, vs. Eri80 group).

Rapamycin was injected into the left brain of MCAO rats to investigate the protective mechanism of eriodictyol. Compared with the MCAO group, rapamycin treatment increased the expression of LC3. The increase of LC3 in the rapamycin group suggested that autophagy was induced by rapamycin. When compared with the MCAO group, the positive rate (red) of LC3 in the treatment with eriodictyol group was reduced (Figure 6A). Compared with the eriodictyol group, the positive rate of LC3 in treatment with eriodictyol and rapamycin was increased. Also, the conversion of LC3 protein, Bax, Bcl-2, cleaved-caspase-3, and Ki67 in different groups was determined via western blotting assay. The results showed that the conversion of LC3, Bax, and cleaved-caspase-3 in eriodictyol group was lower compared with the MCAO group, while the expression of Bcl-2 and Ki67 were increased. And the effects of eriodictyol on the expression of LC3, Bax, Bcl-2, Ki67, cleaved-caspase-3 were reversed by rapamycin (Figure 6B). Further research showed that rapamycin could reduce the level of IL-10 and increase the level of TNF-α (Figure 6C). Treatment with rapamycin also represses the effects of eriodictyol on the levels of IL-10 and TNF-α in MCAO rats. Taken together, the data demonstrated that eriodictyol ameliorated MCAO-induced brain injury by reversing the autophagy dysfunction, affecting apoptosis and inflammation.

## Discussion

Due to high morbidity and mortality, ischemic cerebrovascular disease has gradually become a serious threat to human health worldwide. A large body of evidence has suggested cerebral ischemia may lead to neuronal loss and apoptosis. Accordingly, alleviating apoptosis in the brain has emerged as a new effective strategy to treat ischemic brain diseases.

Eriodictyol, a flavonoid compound isolated from Chinese herb (Dracocephalum rupestre), has anti-inflammatory (Lou et al., 2012), anti-cancer (Zhang et al., 2020b), and antioxidant effects (Bernaudin et al., 1999). As previously reported (Xie et al., 2019) that eriodictyol can significantly improve the vitality of H9c2 cells, inhibit intracellular Ca^2+^ overload, prevent the production of ROS, and regulate the expression level of apoptosis-related proteins to play a cardiac protective role. Ferreira et al. (2016) found that the oral administration of eriodictyol improved neurological function in ischemic stroke rats. Thus the effect of eriodictyol on MCAO-induced brain injury still needs further study.

The purpose of our study was to evaluate the effects of eriodictyol on MCAO-induced brain injury and memory impairment and to explore its brain-protective mechanism. Our results showed that eriodictyol could reduce the infarct size and improve the ability of learning and memory. In addition, we explored the effect of eriodictyol on neuronal apoptosis, and the results showed that eriodictyol could significantly down regulate the expression levels of apoptosis-related signaling proteins such as Bax and cleaved-caspase-3. Furthermore, we found that eriodictyol could inhibit the inflammatory response and suppress autophagy in MCAO rats.

A large number of evidence suggested that Chinese herbs, such as eriodictyol could protect the cell from injury by activating the Nrf2/ARE pathway (Lou et al., 2012). Several flavonoids have been shown to have neuroprotective effects on the activation of astrocytes in rats with ischemic strokes, such as hesperidin, which inhibits neuronal apoptosis and inflammatory response. Similarly, we also found that eriodictyol inhibited neuronal apoptosis and neuro-inflammatory response to play a neuroprotective role in MCAO rats.

Autophagy is a highly regulated lysosomal metabolic decomposition process, which mainly involves a variety of pathophysiological conditions, especially I/R injury. Autophagy has been considered a double-edged sword with prosurvival or prodeath potential in cerebral I/R injury, which may promote cell survival but also promote neuronal apoptosis through excessive self-digestion and degradation of essential cellular constituents (Aghaei et al., 2019). Jiang et al. (2017) demonstrated that autophagy was overactivated in MCAO rats or OGD/R PC12 cells, and NaHS inhibition of autophagy could alleviate ischemia/reperfusion injury *in vivo* and *in vitro* (Jiang et al., 2017). NMDA receptor antagonists can inhibit autophagy and alleviate ischemia-reperfusion injury (Dong et al., 2017). On the other hand, rapamycin, as an autophagy stimulus, relieved cerebral ischemia-reperfusion injury. Rapamycin could inhibit the activation of mTOR pathway in the MCAO rats, activate autophagy, suggesting that rapamycin has a protective effect on cerebral I/R injury (Wu et al., 2018). Recent studies have indicated that eriodictyol might regulate autophagy in neurodegenerative diseases. Here, we assess the autophagy flux by detecting the molecular markers of autophagy during MCAO induced brain injury. Studies have shown that autophagy accelerates progress in the presence of ATG5 (Shi et al., 2018). Beclin 1 plays an important role as a regulator by modulating both autophagy and apoptosis (Ma et al., 2016). Additionally, p62 acts as a cargo receptor for the autophagic degradation of substrates. In the study, we found that the level of LC3, ATG5, and Beclin-1 in the MCAO group was significantly increased, whereas the expression of p62 was reduced. After treatment with eriodictyol, the level of LC3, ATG5 and Beclin-1 was reduced significantly, whereas the expression of p62 was increased markedly. Compared with apoptosis and inflammation, rapamycin activated autophagy markedly in MCAO rats, which may be related to the regulation of Beclin1 and rapamycin. As shown in Figure 6, we found that rapamycin could significantly up-regulate the expressions of autophagy markers (LC3) inhibited by eriodictyol in MCAO rats. Rapamycin could reverse the inhibitory effect of eriodictyol on autophagy, suggesting that suppression of autophagy plays a dominant role in the neuroprotective effect of eriodictyol. Our findings offer more details, confirming that eriodictyol attenuated I/R injury by inhibiting autophagy in MCAO rats.

In addition to autophagy, there are a large number of inflammatory cytokines release and neuronal apoptosis, and other pathological processes in cerebral ischemia-reperfusion injury. It has been (Bai et al., 2019) reported that eriodictyol could suppress the activation of Akt/NF-κB to inhibit inflammation and oxidative stress in HG-induced human glomerular mesangial cells. Eriodictyol can improve LPS-induced inflammatory response and oxidative stress by inhibiting MAPKs and NF-κB pathways and balancing Nrf2/KeAP1 antioxidant defense pathway in BV-2 microglial cells and mouse brain (He et al., 2019). Similarly, our results also confirmed that eriodictyol can regulate the release of inflammatory factors and inhibit the outbreak of inflammatory response in cerebral I/R injury. To further elucidate the protective effect of eriodictyol on ischemia-reperfusion injury, TUNEL staining and western blot results showed that eriodictyol inhibited the expression of apoptosis-related proteins such as Bax and cleaved-caspase-3, and promoted the expression of Bcl-2. And as shown in Figures 3A–C, the immunohistology staining results showed that eriodictyol inhibited neuron loss and promoted proliferation.

In summary, in the present study, we found eriodictyol could inhibit the neuronal loss and apoptosis of brain tissues in MCAO rats. Meanwhile, eriodictyol directly affected the autophagy of neurons in MCAO rats and played a momentously protective role. Additionally, eriodictyol pretreatment prevented the increases in TNF-α and IL-6 and the down-regulation of IL-10 and IL-β1 in serum and cerebral cortex of MCAO rats. It suggested that eriodictyol can reduce the inflammation response of brain injury lesions and inhibit neuroapoptosis and promote proliferation, which may be partly attributed to the reversal of the protective effect of autophagy.

## Data Availability Statement

The raw data supporting the conclusions of this article will be made available by the authors, without undue reservation.

## Ethics Statement

The animal study was reviewed and approved by Hubei Provincial Hospital of Traditional Chinese Medicine.

## Author Contributions

CW: writing, conceptualization, methodology, and software. ZM: data curation, writing, and original draft preparation. ZW: visualization and investigation. SM: supervision and methodology. YD: software, validation, and data curation. SZ: investigation. HQ: reviewing and editing. All authors contributed to the article and approved the submitted version.

## Conflict of Interest

The authors declare that the research was conducted in the absence of any commercial or financial relationships that could be construed as a potential conflict of interest.

## Supplementary Material

SUPPLEMENTARY FIGURE 1 Effect of eriodictyol on blood flow in MCAO rats.
