# Supplementary figures and images for: Eriodictyol Attenuates MCAO-Induced Brain Injury and Neurological Deficits via Reversing the Autophagy Dysfunction
Source: Front Syst Neurosci. 2021 May 26;15:655125. doi: 10.3389/fnsys.2021.655125 (PMC8190663; doi:10.3389/fnsys.2021.655125)

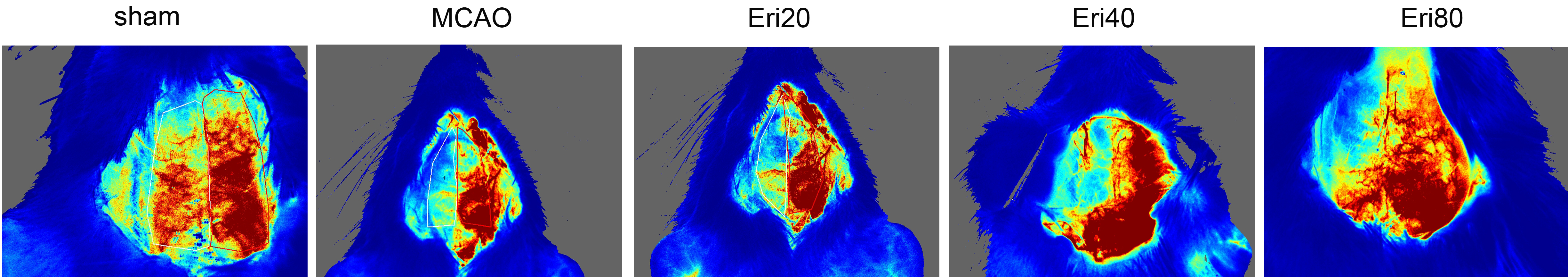

Supplement: SUPPLEMENTARY FIGURE 1 — Effect of eriodictyol on blood flow in MCAO rats. [file Image_1.JPEG]
